# Isolation of Exopolysaccharide-Producing Yeast and Lactic Acid Bacteria from Quinoa (*Chenopodium Quinoa)* Sourdough Fermentation

**DOI:** 10.3390/foods9030337

**Published:** 2020-03-13

**Authors:** Wendy Franco, Ilenys M. Pérez-Díaz, Lauren Connelly, Joscelin T. Diaz

**Affiliations:** 1 Departamento de Ingeniería Química y Bioprocesos, Escuela de Ingeniería, Pontificia Universidad Católica de Chile, Ave. Vicuña Mackena 4860, Santiago 7820436, Chile; 2 Departamento Ciencias de la Salud, Carrera de Nutrición y Dietética. Facultad de Medicina, Pontificia Universidad Católica de Chile, Ave. Vicuña Mackena 4860, Santiago 7820436, Chile; 3 U.S. Department of Agriculture, Agricultural Research Service, SAA Food Science Research Unit, 322 Schaub Hall, Box 7624, North Carolina State University, Raleigh, NC 27695, USA; ilenys.perez-diaz@usda.gov; 4 Department of Food, Bioprocessing and Nutrition Sciences, 400 Dan Allen Drive, North Carolina State University, Raleigh, NC 27696, USA; lconne2@ncsu.edu (L.C.); jtdiaz@ncsu.edu (J.T.D.)

**Keywords:** lactic acid bacteria, pseudocereals, fermentation, quinoa, exopolysaccharides

## Abstract

Quinoa, a nutritional grain, can be used as an ingredient in gluten-free sourdoughs. This study characterizes quinoa flour spontaneous fermentation with emphasis in the isolation of exopolysaccharide (EPS) producer bacteria. Real, red and black grains were studied. Dough yield, microbiota composition and fermentation biochemistry were determined for a total of 36 quinoa flour fermentations. The fermentation biochemistry was monitored by high-performance liquid chromatography (HPLC) analysis, pH measurement and titratable acidity. Changes in the microbiota were monitored by plating on deMann Rogosa and Sharp 5 agar (MRS5) and yeast and mold agar (YMA) plates and with metagenetic analysis. The ability to produce exopolysaccharides was screened in selected lactic acid bacteria (LAB) isolates. Production of organic acids in the spontaneous fermentation dropped the pH to 4.0 ± 0.3. The community of presumptive LAB reached 8.37 ± 0.01 log colony forming units (CFU)/mL by day 8 of back-slopped fermentations. The microbiota was composed of *Lactobacillus*, *Enterococcus, Leuconostoc*, *Lactococcus*, *Pediococcus* and *Weissella*. *P. pentosaceous,* *L. citreum* and *W. cibaria* were able to produce EPS in a starch-rich medium. *P. pentosaceous* showed higher exopolysaccharide yield, rapid acidifying kinetics and was able to drop the dough broth pH to values below 4.0 and a positive fermentation quotient after 24 h of incubation. Therefore, the bacterium might be a potential candidate for quinoa sourdough production.

## 1. Introduction

Originally from the Andes, quinoa (*Chenopodium quinoa*) is a seed crop rich in proteins, lipids, fiber, vitamins and minerals. Its protein content (approximately 14% *w*/*w*) is superior to wheat and other cereals [1,2], and it is characterized for its excellent balance of essential amino acids. Moreover, quinoa has been found to contain other bioactive compounds including saponins, phytosterols, phytoecdysteroids, phenolics, betalains, glycine betaine and isoflavones [1,2]. In addition, quinoa seeds are gluten-free (GF), and therefore an ideal ingredient for the diet of celiac patients.

Celiac disease (CD) is a chronic enteropathy trigged by the ingestion of gluten. No pharmacological treatment is available to CD patients, and the only alternative is a life-long avoidance of gluten-containing food products. This is a challenge that is difficult to accomplish, mainly because cereal foods, rich in gluten in various forms, are an essential component of the daily diet (i.e., bread). Moreover, they also provide several important nutrients [3]. Therefore, a gluten-free (GF) diet may lead to nutritional deficiencies [4,5,6].

Even though quinoa is an alternative for GF diets, its use in bread-making has been limited because of its low baking quality [7]. An alternative to overcome these problems is to use lactic acid fermentation as part of the bread-making process. Sourdough (SD) technology has been successfully used to improve the flavor and texture of other GF-cereal breads, and can be applied to the quinoa flour as well [8,9]. Among other applications, quinoa flour (alone or as sourdough) has been added to gluten-containing bread formulations with the intention of improving its nutritional, textural and/or sensorial profile [6,10,11,12,13]. Rizzello et al. (2016) reported that the addition of 20% (*w*/*w*) quinoa sourdough to wheat breads, resulted in better chemical, textural, and sensory features than wheat bread alone or with quinoa flour (not fermented) as an additive [13].

Exopolysaccharides (EPSs) of microbial origin have potential applications as texturizers, viscosifiers, emulsifiers and syneresis-lowering agents due to their pseudoplastic rheological behavior and water-binding capacity [14]. Many food-grade microorganisms produce EPS [15,16] and have been studied as natural alternatives for the production of plant-based polysaccharides. Recently, EPS-producing bacteria have gained attention for application in bakery products. Fructan from *Lactobacillus sanfransiscensis* has been reported as positively influencing dough rheology and bread texture [17,18,19]. According to Brandt and Bode (2009), EPS in situ production is more suitable for bread formulation than the addition of the same polysaccharide into the formulation or processing [20].

Even though the impact of EPS production in wheat and rye sourdoughs have been studied and described [21,22], its functions in GF formulations remain undocumented. The few studies available have demonstrated that stalling is prevented, and that crumb volume and softness is improved. Softer crumbs were reported when SD technology was applied to the formulation of sorghum bread [23,24], and loaf specific volume and crumb texture increased in oat bread [25].

Although, most lactic acid bacteria (LAB) used for sourdough bread production have been isolated from gluten-containing flours [17,22], some studies have reported autochthonous LAB species associated to GF-free cereal fermentations. A broad of spectrum of LAB were identified as belonging to the genera *Lactobacillus, Pediococcus, Leuconostoc*, and *Weisella* [9,25,26,27]. The fitness of some isolates were tested to improve sensorial and nutritional attributes of sourdough bread [6,13]; however, to our knowledge little has been investigated on EPS-producing bacteria associated to quinoa sourdough production. In situ formation of bacterial hydrocolloids might have a great impact on the rheological attributes for bread made only with GF-free flours, in which the lack of gluten is a rheological problem. In order to identify and select potential starter candidates it is necessary to evaluate its technical capabilities and, therefore, the objectives of this study were to characterize the indigenous microbiota in three types of quinoa flour (real, red and black) sourdough fermentation; focused on the isolation and characterization of EPS-producing bacteria, and to evaluate its fermentation fitness.

## 2. Materials and Methods

### 2.1. Quinoa Grains

Four different types of quinoa grains including real, red and black cultivated at “Los Lipez” (Potosí, Bolivia, 20°44′24″ S, 67°39′42″ O) were purchased in stores located in La Paz, Bolivia, while mixed grains cuktivated at Isla Grande de Chiloé (Region de Los Lagos, Chile, 42° 40′ 36″ S 73° 59′ 36″ O) were purchased in Santiago (Chile). Two lots of each quinoa grain product were secured. Quinoa grains were desaponified, and milled into wholegrain flour (Foss, Cyclotec 1093 Mill, Copenhagen, Denmark). The flours were stored in sterile containers at 4 °C until use. Flours proximal composition was determined following the Official Methods of Analysis [28].

### 2.2. Spontaneous Quinoa Flour Fermentation

Flour and deionized (DI) water were mixed using a 50:50 (*w*/*v*) ratio in sterile containers until a homogenous mixture was formed. Dough yield was determined by:

(1) $$\mathrm{dough}\;\mathrm{yield}=\frac{\mathrm{dough}\;\mathrm{mass}}{\mathrm{Flour}\;\mathrm{mass}}*100$$

**Figure 1:**
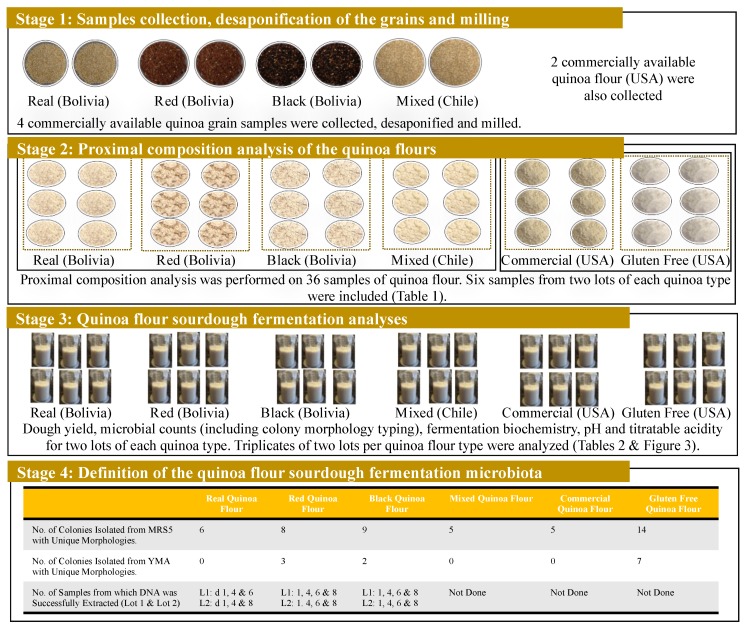
Flow diagram summarizing the research approach applied in this study for the quinoa spontaneous sourdough fermentation.

The mixture was fermented at 30 °C to allow the establishment and growth of the indigenous microbiota under static conditions. Back-slopping was performed with 1% of the ripe sourdough every 24 h following the standard protocol for type 1 sourdough making [29]. The procedure was repeated for 8 d, a time at which a stable microbiota was observed. A stable microbiota was defined as the appearance of approximately the same number of colony forming units (CFUs) on culture plates with similar morphology characteristics. A portion of ripe sourdoughs were used for the chemical and microbiological analyses, and another portion was stored at −20 °C for further testing. Triplicate samples of each lot were analyzed. Two lots of each quinoa flour type (3) were tested accounting for a total of 16 samples (Figure 1).

#### 2.2.1. Chemical Analysis of the Quinoa Dough Fermentation Samples

The hydrogen ions concentrations in every sample were measured using an Accumet pH-meter (Accumet^®^ Research 25 pH meter, Fisher Scientific, Carlsbad, CA, USA) equipped with Gel-Filled Pencil-Thin pH Combination Electrodes (Accumet Fisher Scientific, Pittsburgh, PA, USA). Titratable acidity was calculated based on the amount of 0.1 M NaOH (mL, Sigma-Aldrich, Saint Louis, MO, USA) needed to achieve a pH of 8.5 [25]. Concentrations of sugars, primarily glucose and fructose, and organic acids were determined by high-performance liquid chromatography (HPLC) as described by McFeeters and Barish (2003) [30]. Samples were prepared for HPLC analysis as described by Ampe et al. (1999) [31] and Lattanzi et al. (2013) [32]. Triplicate samples of each lot were analyzed. Two lots of each quinoa flour type (6) were tested accounting for a total of 36 samples (Figure 1).

#### 2.2.2. Identification and Characterization of the Fermented Quinoa Sourdough Microbiota

Collected samples were plated in deMann Rogosa and Sharp 5 Agar (MRS5) and yeast and mold agar (YMA) for LAB and yeast, respectively. Colonies in plates were grouped according to morphology. Four to five colonies presenting unique morphologies in both MRS5 and YMA plates were collected on day 8 and isolated among the six replicates for each quinoa flour type. Colonies were streaked for purification in the respective culture solid medium for further characterization and identification. Frozen stocks were prepared in MRS5 or YMA broth supplemented with 15% glycerol (Cat No. G5516, Sigma, St. Louis, MO, USA) for LAB and yeasts, respectively.

The bacterial and yeast isolates were identified using the partial sequence of the 16S or 26S rDNA, respectively. Chromosomal DNA was extracted using the InstaGene DNA isolation matrix (Instagene, BioRad, Hercules, CA, USA) and MasterPure™ Yeast DNA Purification Kit (Epicentre Biotechnologies, Madison, WI, USA) for bacteria and yeast, respectively. Bacterial DNA was amplified by polymerase chain reaction (PCR) with the primer pair 27F/1492r [33] and yeast DNA with NL-1/NL-4 (Kurtzman and Robnett, 1997) [34]. All primers were obtained from Integrated DNA Technologies (Coralville, IA, USA). PCR products were sequenced by Eton Bioscience Inc. (Raleigh, NC, USA). The sequences obtained were subjected to the basic local alignment search tool (BLAST 2.2.26) [35] in GenBank [36] using the 16S rDNA microbial database for the bacterial cultures and the non-redundant nucleotide database for the yeast cultures [33] for identification. Sequences can be found in the National Center for Biotechnology Information (NCBI)-GenBank with accession numbers MH544758-MH544805 for the bacterial isolates and MH544826-MH544837 for the yeasts cultures.

#### 2.2.3. Preparation and Sequencing of the Quinoa Dough Fermentation 16S rDNA Library

Cells harvested from 10 g of fermented quinoa flour were resuspended in sterile saline and treated with 2.5 mM propidium monoazide (PMA) stock solution (1.3 mg/mL PMA in 20% DMSO; Biotium, Inc., Hayword, CA, USA) to eliminate dead bacterial and extracellular DNA as described by (Pan and Breidt, 2007). PMA-treated samples were stored as cell pellets at −20 °C until DNA extraction was performed. Total genomic DNA was extracted using a MasterPure^TM^ DNA Purification Kit (Epicentre, Madison, WI, USA). The 16S rDNA gene regions V3 to V4 were amplified by PCR using the Bakt_341F/Bakt_805R primers [37]. Primers were barcoded as described by the manufacturer for the Illumina MiSeq sequencing technology. For the fungi analysis, primers ITS1F/ITS2 were used. Sequencing services were obtained from the Carver Biotech Laboratory at the WM Keck Center for Comparative and Functional Genomics (Chicago, IL, USA). Twenty-two samples corresponding to 3 quinoa flour-type fermentations including real, red and black collected at different time points (day 1, 6 and 8) were subjected to DNA extractions and sequencing data processing.

#### 2.2.4. Processing of the 16S rDNA Amplicon Sequences Data Corresponding to Quinoa Dough Fermentation Samples

The reads from fermentation samples were quality-trimmed with Trimmomatic (version 0.36), and any reads with lengths less than 36 bp were removed [38]. Primer sequences were removed using MacQIIME 1.9.1-20150604 [39]. The reads were merged using fastq-join with the default parameters [40], and merged reads with Phred quality scores less than 20 were removed. VSearch [41] was used to de-replicate the sequences and remove singletons, sort on sequence abundance and cluster the sequences at 97%. The most abundant read from each cluster was selected as the cluster centroid and, with VSearch, chimeras were removed from the list of centroids. The remaining sequences served as a set of representative sequences. The read sequences were mapped to the representative sequences to generate the orthologue taxonomic units (OTUs) table. Taxonomy was assigned to the OTUs using SILVA (version 128) database [42,43]. The OTUs that represented less than 0.005% of the total sequences were removed, along with those that corresponded to chloroplasts and/or mitochondria. MacQIIME 1.9.1-20150604 was used to calculate the alpha diversity metrics including phylogenetic diversity (PD), Chao1, and the observed OTUs count to assess the sampling depth. PYNAST was used to align the sequences using the SILVA (version 128) core alignment sequences [44]. Based on the resulting alpha diversity plots, a rarefaction level of 20,000 sequences per sample was selected. Sequencing data is available from the National Center for Biotechnology Information.

#### 2.2.5. Processing of the ITS Amplicon Sequences Data Corresponding to Quinoa Dough Fermentation Samples

For the fungi analysis, reads below 36 bp were removed from the pool of sequences to be analyzed. Forward and reverse reads were merged with a minimum overlap of 50 bp and reads with quality scores less than 20 and 200 bases in length were removed. Trimming of the reads was accomplished with an ITS Extractor (ITSx; Bengtsson-Palme et al., 2013). Data was processed as described above for the bacterial analysis. However, taxonomy assignments were done using the UNITE database (version 20 November 2016, https://unite.ut.ee/repository.php). OTUs that had less than 0.005% of the total sequences were excluded. The sequences for the amplicons of the ITS regions corresponding to the real quinoa flour and the first two-time points for the red quinoa flour (d 1 and 4) were excluded from the OTUs table given that less than 1000 reads were detected. Samples were rarefied to 7000 reads for the Beta and Alpha Diversity analyses for samples containing more than 1000 reads.

#### 2.2.6. Identification and Characterization of Exopolysaccharide (EPS)-Producing Microorganisms

The screening of EPS-producing microbes was conducted as described by Ruiz-Rodriguez et al. (2016) with some modifications [9]. Briefly, active cultures of bacteria and yeasts were streaked on MRS5 and YMA agar plates, respectively. The media were supplemented with 2% of the following carbon sources: glucose, fructose, sucrose, lactose, and soluble starch. Pure cultures were incubated at 30 °C (bacteria) or 25 °C (yeast) for 2 to 7 d. Colonies presenting slimy surfaces and ropiness to the touch with a loop were reported as EPS-producing [9]. In addition, the isolates were transferred to broth with the afore mentioned carbon sources and incubated for 24 h at 30 or 25 °C for bacteria and yeast, respectively. After incubation, cells were removed by centrifugation (12,000 rpm, 15 min) and EPS precipitated by the addition of one volume of cold ethanol (95%). EPS was recovered by centrifugation and dried to determinate the yield by weigh.

### 2.3. Selection of Isolates with Potential as Starter Cultures for Quinoa Flour Sourdough Fermentation

The fermentation potential of selected bacterial isolates was evaluated in quinoa flour liquid broth prepared as described by Alfonzo et al. (2013) [45]. Briefly, 200 g of quinoa real flour (La Paz, Bolivia) was suspended in 1 L of distilled water and sterilized at 121 °C for 20 min. The flour was removed by precipitation and the supernatant was used as the fermentation broth [45]. LAB cells (overnight) were harvest from MRS5 broth by centrifugation, after 18–24 h of incubation at 30 °C. The pellets were washed twice with peptone water (0.1%) and suspended in the same solution. The inocula were transferred to the fermentation broth to 5 log CFU/mL and incubated for 18 h at 30 °C. Samples were collected and analyzed for pH, Tritable acidity (TTA), lactic and acetic acid concentrations, and colony counts after 8 and 18 h of incubation.

### 2.4. Statistical Analysis

Experiments were carried out in triplicate for two independent assays. Resulting data were analyzed using the analysis of variance (ANOVA) procedure with the Duncan’s multiple range test of the Statistical Analysis Systems version 9.0 (Statistical Analysis System, SAS Institute, Cary, NC, USA).

## 3. Results and Discussion

### 3.1. Spontaneous Quinoa Sourdough: Chemical Characterization

Although, quinoa real is the most common and commercially available grain, there are other colored varieties that have gain attention in the last few years, such as the red and black. The three varieties were studied (Figure 1). The proximal composition of the quinoa flours tested presented insubstantial variations and were similar to those previously reported [1,2] (Table 1).

Starch was the predominant carbohydrate on each flour (>36%), while fermentable sugars represented the lower concentration (about 2%). Black and red grains were higher in crude fiber content (cellulose and lignin). Dough yields during fermentation were in the range of 201 ± 6.3 to 203.7 ± 6.0% (Table 2), which agrees with reports for traditional sourdough fermentations [46].

As expected, the dough pH decreased as a function of time during fermentation achieving an average value of 4.0 ± 0.3 for the three quinoa flours (Table 2).Although, drastic changes in pH were observed for the first 3 d of fermentation with a 2 pH units drop (from 6.5 initial values), no significant differences in pH were recorded after that day (data not shown). While, the pH values achieved for the quinoa flours are slightly higher in comparison to sourdough wheat fermentations [46], they are similar to other GF pseudocereals, such as amaranth [27] and quinoa [13]. In correlation with the pH changes, it was possible to observe increases in titratable acidity as the fermentation developed (Table 2). The changes in pH to 4.1 ± 0.1 and titratable acidity to 17.0 ± 2 occurred concomitantly with lactic acid and acetic acid production (Table 2). In non-fermented quinoa flour the TTA was approximately 1.75 ± 1.12 and lactic or acetic were below the detection limit (data not shown). The production of lactic and acetic acids to equal concentrations is associated with heterofermentations, characteristic of the traditional sourdough fermentation process [47].

### 3.2. Carbohydrate Use during Spontaneous Quinoa Sourdough

The changes in carbohydrate composition during the spontaneous fermentation was also studied. In quinoa flours, fermentable sugars and starch represented 2% and 36%–38% of total carbohydrate, respectively (Table 1), which is in line with previous publications [48]. Once the back-slopped fermentation stopped, fermentable sugars were not detected, while starch consumption was approximately 45% depending on the quinoa flour type. Besides the effect given by the acidification process, LAB used in sourdough fermentation are capable of contributing to starch utilization, particularly the heterofermentative species [47]. Rizzello et al. (2016) reported that white bread manufactured with quinoa sourdough starter (*P. pentosaceous, Lb. plantarum*, *Lb. rossiae*) showed a lower glycemic index, which was associated with starch fermentation by homo- and heterofermentative LAB. In our study a predominantly heterofermentative microbiota was isolated that might be associated with starch utilization. Moreover, the LAB capable of producing EPS when starch is the carbon source were of the heterofermentative types (*P. pentosaceous*, *L. citreum*, and *W. cibaria*, Table 3).

### 3.3. Bacteria in Spontaneous Quinoa Sourdough

**Figure 2:**
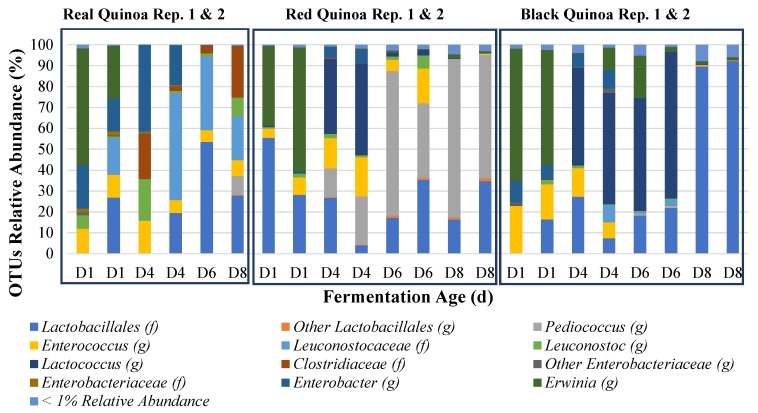
Results of the 16S rRNA gene amplicon sequencing data analysis for quinoa-flour-sourdough samples collected on days 1, 4, 6 and 8 of the fermentation. Data presented by quinoa flour type (real, red and black) sampled for 8 days during the back-slopped spontaneous fermentation with replicate 1 (lot 1) shown first followed by independent replicate 2 (lot 2). Data for the real quinoa replicates 1 and 2 samples were not obtained for D8 and D6, respectively. The letter D followed by a number represents sample day.

Quinoa flour fermentations were dominated by LAB (homo and hetero fermentative) and a few yeasts in parallel to the typical sourdough fermentations. The changes in the bacterial population were mainly in regards to the reduction in biodiversity (Figure 2) as expected from the application of food preservation techniques, such as fermentation.

A few LAB genera prevailed in the fermentations including, in order of dominance, *Lactobacillus*, *Leuconostoc*, *Enterococcus*, *Lactococcus*, and *Pediococcus* (Figure 2). Quinoa real flour fermentation showed the greatest diversity in the microbiota after 8 days (Figure 2). Approximately 80% of the bacterial population in real quinoa doughs were LAB belonging to the *Lactobacillates* and *Leuconostoccacea families* (Figure 2). In red quinoa flour fermentation *Pediococcus* was the predominant genus, followed by *Lactobacillus* (Figure 2). *Lactobacillus* was the predominant genera in black quinoa flour fermentation (Figure 2). Variation in macro-elements composition among grains and flours may influence the establishment of microbial communities during sourdough spontaneous fermentations. It has been reported that the adaptability and dominance of selected microbial strains is closely related to the nutrients provided by their habitat [47,49].

*Lactococcus* spp. were primarily detected between day 4 and 6, particularly in the red and black quinoa flour samples with a substantial relative abundance as compared to *Pediococcus* spp. (Figure 2). The red and black quinoa varieties had the higher ash and fiber contents (Table 1). According to De Vuyst et al. (2014), the origin and composition of the soil influences the establishment of microbial communities in sourdough fermentations [47]. Further research is needed to determine if the ash and fiber content in quinoa grains predisposes a fermentation to the prevalence of lactococci.

*Erwinia* and *Enterobacter* spp. are abundant on the first few days of fermentation, but such bacteria were not detected as a function of acidification and time (Figure 2). Similar results were reported by Sterr et al., 2009, in which enterobacteria present at the beginning of the amaranth flour fermentation were rapidly controlled after 1 day of fermentation [27]. However, the real quinoa flour fermentation showed the presence of clostridial species to 5% and 25% relative abundance on replicates 1 and 2, respectively, at day 8 of the fermentation (Figure 2), suggesting that a suboptimal fermentation could compromise the safety of the dough. However, pH values at this time point were below 4.6 which is inhibitory for the growth of *Clostridium botulinum*, the only pathogen in this genus (Table 2). No clostridiales were observed in the red or black quinoa flour fermentations at day 8.

The culture dependent analysis showed that colony counts from MRS5 plates reached 8.34 ± 0.1 log CFU/g, representing a 4 log increase from the initial counts at 4.5 log CFU/g, independent of the quinoa variety (Table 2). Twenty-three colonies were isolated from MRS5 plates inoculated with 8 d old quinoa flour fermentation samples. Such colonies presented variable morphological features and were identified as LAB (Table 3).

**Figure 3:**
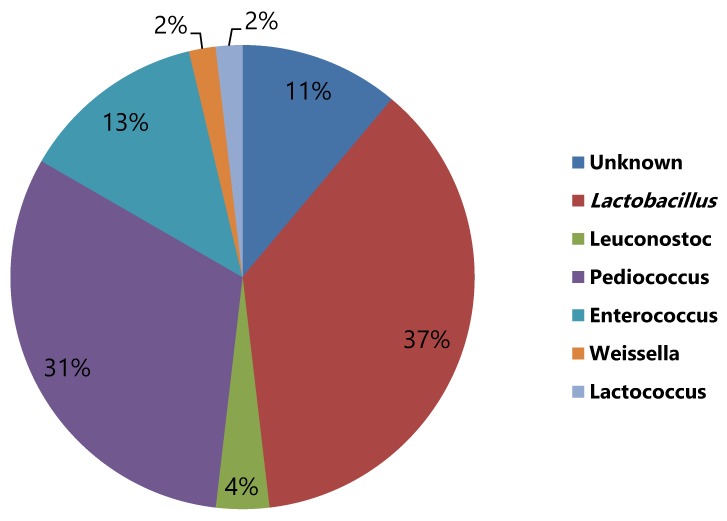
Estimated distribution of lactic acid bacteria species in the 18 quinoa flour fermentations tested based on colony morphology. Colony were grouped accordingly to morphology. Clones were sequenced and identified by 16 sRNA.

Identification of the isolates by 16S rDNA gene sequencing showed that the dominant LAB in quinoa flour fermentations after 8 d back-slopping are, in order of prevalence, *Lactobacillus* (29%), *Pediococcus* (29%), *Enterococcus* (25%), *Leuconostoc* (9%), *Weissella* (4%) and *Lactococcus* (4%) (Table 3, Figure 3). These results are in line with those obtained from the culture-independent testing described above (Figure 2). Table 3 describes the specific species present in the fermentations using culture dependent techniques as *Lb. plantarum*, *Lb. paraplantarum*, *Lb. sakei, Lb. graminis, Lb. sakei, P. pentosaceous*, *E. faecium*, *E. mundtii*, *W. cibaria*, *Le. mesenteroides* and *L. lactis*. The diversity observed in this study is in line with that previously published for cereal fermentations [16,45,49,50,51] and gluten-free flours [9,13,25,27,49].

### 3.4. EPS-Producer Isolated Bacteria

From all the bacterial isolates screened, only the *Pediococcus*, *Leuconostoc* and *Weissella* spp. were able to produce EPS from starch (Table 4).

All isolates, except *Lb. graminis*, *Lb. paraplantarum,* and *Lb. sakei,* were able to produce EPS with sucrose as the carbon source, and only *P. pentosaceus, Lb. plantarum* and *L. lactis* showed production from fructose. *P, pentosaceous*, *L. lactis* and *W. cibaria* were able to produce EPS from lactose (Table 4). Among all the isolates tested, only the three *Pedioccocus* species showed EPS production with the four different carbon sources. In addition, these isolates showed greater yields (2%) when tested in sucrose, fructose and starch (Table 4).

EPS have been associated with the improvement of the organoleptic properties of sourdough and its incorporation in quinoa flour fermentation brings an opportunity to optimize rheology attributes in GF-free doughs [16,18].

EPS producing bacteria are known to be fastidious growers, aciduric, and enhancers of bread attributes (Lazaridou et al., 2007). Its establishment is determine by the type of carbohydrate available, pH and the capability to outcompete other microorganisms, including yeast [52]. Therefore, the establishment of EPS-producing LAB comes with a technological advantage for quinoa sourdough bread making.

### 3.5. Fermentative Potential of Selected EPS-Producer Isolates

Pure colonies were inoculated in sterile quinoa fermentation broth (QFB) to characterize the fermentation potential of each of the species identified, particularly the EPS-producing cultures. A sterile fermentation broth was chosen over the commonly used non-sterile doughs to understand the specific contribution of the isolated bacteria to the fermentation biochemistry and the growth profile in a defined substrate. Seven out of the 15 strains tested in quinoa fermentation broth acidified the media to pH values below 4.0 when the fermentation proceeded for 18 h (Table 5).

Similar acidification profiles were reported by Moroni, Arendt, and Dal Bello (2001) when following the spontaneous buckwheat and teff sourdough fermentations [26]. *E. faecium, Le. citreum, Le. mesenteroides, P. pentosaceous* and *W. cibaria* were able to decrease the dough pH below 5.0 after 8 h, while *W. cibaria*, *P. pentosaceous* and *Le. citreum* strains were able to decrease it to values below 4.6 (Table 5). The TTA data collected from the quinoa fermentation broth samples confirmed the observations made with respect to changes in pH. *Weissella* and *Leuconostoc* also presented a rapid kinetic of acidification in sterile and non-sterile wheat broth fermentations [45]. *P. pentosaceous* strains can also produce oat dough with a pH of 4.36 and TTA of 10.8 [25]. The microbial counts observed correlated with pH and TTA measurements; with increasing colony counts corresponding to higher TTA and lower pH after 8 and 18 h of incubation (Table 5). The increment in colony counts observed in this experiment are lower than those reported for non-sterile quinoa flour doughs after 16 h of incubation [12,13]. An increase in colony counts of 1.5 log CFU/g was observed in sterile wheat dough after 21 h of fermentation for *Le. citreum*, *Le. mesenteroides* and *W. cibaria* [45].

The inoculation of *L. lactis*, *Lb. sakei*, *Lb. graminis*, *Lb. paraplantarum* and *Lb. plantarum* in quinoa fermentation broth resulted in minimal changes in pH to values greater than 5.0, produced lower TTA, an experienced a decline in colony counts as a function of time after 8 h of fermentation and were below detection limits by 18 h (Table 5). These observations suggest that although these species may be present in type 1, spontaneous quinoa flour fermentations, they may not be the most robust candidates for starter cultures to be applied in type II or type III fermentations [53].

According to De Vuyst et al. (2014), mostly heterofermentors are commonly found in sourdough fermentation due to the adaptability to the carbohydrate flour characteristics. Conversely obligate homofermentors might have a disadvantage in quinoa flour fermentations [47]. In our study, *P. pentosaceous*, a facultative herterofermenter, was one of the dominant bacterium at the end of the spontaneous fermentation followed by a diversity of lactobacilli. *Pediococcus* can adapt to different carbon sources and amino acid uptakes [47], and therefore might have a competitive advantage that allowed such dominance (Figure 2). Other *Pediococcus* spp. have also been reported as suitable for cereal fermentations given the ability to rapidly acidify and prevail in amaranth flour fermentation enabling stable fermentations at different temperatures [27]. *P. pentosaceous* has been isolated from various cereals [25,26,47] and vegetable fermentations [54]. While its ability to produce EPS in cereal-based beverages has been reported [55], there is a lack of information for sourdough applications.

Table 6 shows the fermentation biochemistry profile for pure cultures of LAB in quinoa fermentation broth. Lactic acid was in the range of 1.32 ± 0.01 to 6.47 ± 0.02 mg/g with the lowest values shown in descending order by *Lb. plantarum*, *Lb. sakei*, *L. lactis* and *Lb. graminis* (Table 6). The lowest acetic acid concentration ranging from 0.25 ± 0.02 to 0.39 ± 0.01 mg/g were observed for *Lb. sakei*, *Lb. paraplantarum* and *Lb. plantarum*. For the other bacteria, the concentration ranged between 0.61 ± 0.05 to 0.87 ± 0.01, with *Lb. graminis* having the highest concentration (Table 6).

The fermentation quotient (FQ) relates to the ratio of lactic acid and acetic acid concentrations produced and was used to determine the potential effects in the aroma and texture profiles of gluten-containing flours. Lactic acid contributes to form a more elastic dough, while acetic acid is responsible for a shorter and harder dough and, therefore, a greater concentration of the first is desirable [50]. Values in the range of 1.5 to 4.0 positively affect the aroma profile and structure of the finished products with optimal values between 2.0 and 2.7 [51,56]. The FQs for the quinoa fermentation broth cultures with LAB isolated from quinoa flour spontaneous fermentations ranged between 3.3 to 9.91. These observations indicate that while 4 isolates met the desired criteria (*W. cibaria, P. pentosaceous, Lb. plantarum,* and *Le. citreum*), none produced an FQ value within the optimal range. Further research is needed to determine if the FQ values applicable to gluten-containing breads are equally meaningful in the GF counterparts and, if so, optimize quinoa flour fermentation to meet such parameters.

### 3.6. Yeast in Spontaneous Quinoa Sourdough

While LAB prevailed in the quinoa flour fermentations, the colonization of yeasts occurred after 6 d of back-slopping (data not shown). Generally, yeasts tend to present longer lag phases and higher doubling times in sourdough fermentations, which enables LAB to exhaust the readily available energy sources and outcompete them [16]. The yeasts commonly present in cereal fermentations are known to associate with LAB for persistence [16]. Total yeast and molds colony counts corresponding to the red quinoa flours were at 5.45 ± 0.3 log CFU/g; while 2-log higher counts were observed for the black quinoa flours (7.21 ± 0.2 log CFU/g) (Table 2). Yeasts were not detected in real quinoa flour fermentations. Thus, total yeast and mold colony counts were also influenced by the type and, possibly, the origin of the quinoa flour.

**Figure 4:**
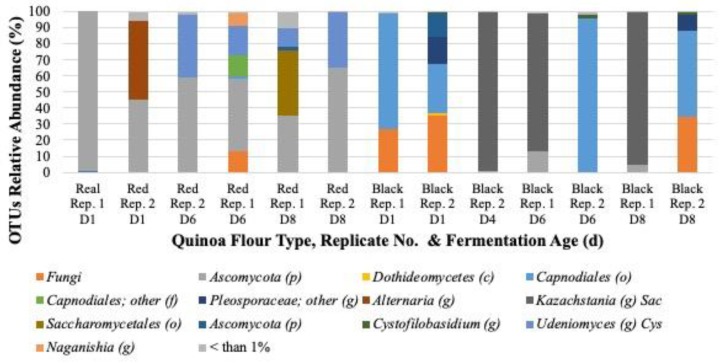
Results of the ITS gene amplicon sequencing data analysis for fermented quinoa flour samples collected on day 1, 4, 6 and 8 of the fermentation. Data presented by quinoa flour type (real, red and black) sampled for 8 days during the back-slopped spontaneous fermentation. The letter D followed by a number represents sample day.

In traditional sourdough fermentations, *Saccharomyces*, *Kazachstania* and *Candida* strains are frequently found, followed by *Pichia, Wickerhamomyces* and *Torulaspora* [16,26,57,58]. Our culture-dependent study revealed the presence of one phylogenetic family: *Saccharomyces* represented by *S. servazzii* (Table 3). The culture independent study revealed a limited diversity in the yeasts population including only 4 genera *Alternaria*, *Udeniomyces*, *Naganishia*, and *Kazachstania* (Figure 4).

Ascomycota were present in red quinoa samples from day 1 to day 8 of back-slopping, decreasing in time for replicate 1, and remained almost unchanged for the second replicate (Figure 4). A succession of yeast types were observed in black quinoa flour fermentations. *Capnodiales* were dominant on day 1. However, as the fermentation proceeded differences were observed among replicates too. *Kazazhstania* were dominant in replicate 1 until day 8, while the replicate 2 showed the prevalence of *Capnodiales* (Figure 4). The difference observed between culture independent and dependent results, might be due to the limitations in the coverage of the ITS sequencing analysis (Supplementary material, Table S1). It is presumed that the analysis was compromised by the lack of diversity within the sequences in the database used for the assignment of OTUs, designed mostly for environmental samples; the diversity of ITS region sequences within the fungi population found in quinoa flour fermentations; and possibly by the technicalities of the genomic DNA extraction and/or PCR protocol used prior to sequencing or a combination thereof. Based on the results it seems that the methodology followed to characterize yeast during quinoa fermentation is not appropriate and it is not in correlation with what has been previously reported. It would be important to further characterize the yeast population in quinoa flour fermentation to understand the possible role and impact in quality. Although, the *Saccharomyces* spp. detected are known to be present in sourdough fermentations, *S. servazii* has not been implicated in cereal fermentations. The yeast isolated tested positive for EPS production, with yields below 1% (Table 4) and were not further tested in this study.

## 4. Conclusions

A stable type 1 quinoa flour fermentation was achieved with a final pH of 4.0 ± 0.1. A biodiverse population of LAB was detected in quinoa flour fermentation along with the yeast *S. servazzi*. Autochthonous EPS-producing LAB such as *P. pentosaceous* showed potential as candidates for starter culture for quinoa flour sourdough fermentation. The autochthonous *P. pentosaceous* demonstrated an ability to grow and compete in quinoa flour dough, a versatile sugar utilization profile and an ability to quickly acidify quinoa fermentation broth and is capable of producing EPS from starch, sucrose, fructose and lactose.

Development of exopolysaccharide-producing starter cultures for quinoa flour sourdough is essential for the elaboration of gluten-free bread with consistent sensory attributes. This study identifies *P. pentosaceous* as a promising exopolysaccharide-producing starter culture for quinoa flour sourdoughs. Further studies should address the behavior of this EPS-producing bacteria in the sourdough making and its contribution in rheological and textural attributes for GF breads.

## Figures and Tables

**Table 1:** Proximal composition of three different varieties of quinoa grains and commercially available quinoa flours with and without gluten.

Flour Type	Humidity	Ash	Carbohydrates	Fermentable Sugars	Starch	Lipids	Proteins	Crude Fiber
	% *							
Red	11.2 ± 0.07 a	2.19 ± 0.05 a	70.1 ± 0.1 a	1.40 ± 0.10 a	37.9 ± 0.52 a	5.47 ± 0.05 a	14.5 ± 0.03 a	6.77 ± 0.17 a
Real	11.9 ± 0.08 a	1.64 ± 0.04 b	68.9 ± 0.4 a	1.37 ± 1.45 a	37.8 ± 0.75 a	3.19 ± 0.03 b	13.7 ± 0.02 b	4.81 ± 0.50 b
Black	11.5 ± 0.38 a	2.17 ± 0.13 a	69.7 ± 0.4 a	1.67 ± 0.99 b	36.5 ± 1.00 b	3.94 ± 0.19 c	14.1 ± 0.12 a	7.03 ± 0.13 a

Values shown correspond to the average of three replicates and two independent trials. Values in a column followed by a different lowercase letter are significantly different (*p* < 0.05).* Dry weight, except for humidity. a–c Values in the same column with different superscript letters differ significantly (*p* < 0.05).

**Table 2:** Dough yield, microbial counts, organic acids, pH and titrable acidity (TTA) observed for spontaneous quinoa flour fermentation samples after 8 days of incubation and daily back-slopping.

Quinoa Flour Type Fermented	Dough Yield (%)	MRS	YMA	Fermentable Sugars %	Starch %	Lactic Acid Produced (mM/Kg)	Acetic Acid Produced (mM)	pH	TTA
		(Log of CFU/g)	(Log of CFU/g)						
Real	203.0 ± 7.9 a	8.40 ± 0.0 a	BDL	BDL	27.9	7.75 ± 0.15 a	4.40 ± 0.13 a	4.11 ± 0.03 a	17.0 ± 1.41 a
Black	203.7 ± 6.0 a	8.37 ± 0.1 a	7.03 ± 0.2 a	BDL	20.8	5.92 ± 0.17 b	5.84 ± 0.11 a	4.12 ± 0.07 a	15.6 ± 0.92 b
Red	201.9 ± 6.3 a	8.35 ± 0.1 a	5.34 ± 0.1 b	BDL	18.25	6.13 ± 0.13 b	4.57 ± 0.15 a	3.93 ± 0.04 b	19.2 ± 1.20 a

Results indicate mean values ± standard deviation of three measurements (carried out in duplicates for two independent fermentations). Those followed by a different lower case letter are significantly different (*p* < 0.05) within the column. BDL: Below detection limit.

**Table 3:** Identification of the lactic acid bacteria and yeasts colonies isolated from day 8 of spontaneous quinoa flour fermentations. Colonies picked represented a unique colony morphology. The colonies isolated were identified based on the 16S rRNA or 26S rRNA sequences for lactic acid bacteria (LAB) and yeasts, respectively.

Flour Type (Total No. of Colonies Isolated)	Bacterial Identification (No. of Colonies)	Theoretical Fermentation Type	16S *rRNA* Sequence Accession Number	Yeast Identification (No. of Colonies)	26S *rRNA* Sequence Accession Number
Red (8 from MRS5 and 3 from YMA)	*P. pentosaceous* (2)	Heterofermentor	MH544761 MH544797	*Saccharomyces servazzii* (3)	MH544759 MH544834 MH544832
	*Lb. sakei subsp. Sakei* (1)	Facultative Heterofermentor	MH544783		
	*E. faecium* (1)	Homofermentor	MH544790		
	*W. cibaria* (1)	Obligate Heterofermentor	MH544791		
	*E. mundtii* (2)	Homofermentor	MH544779 MH544802		
	*Le. mesenteroides* (1)	Obligate Heterofermentor	MH544759		
Black (9 from MRS5 and 2 from YMA)	*Le. citreum* (1)	Obligate Heterofermentor	MH544770	*Saccharomyces servazzii* (2)	MH544828 MH544829
	*Lb. plantarum* (3)	Facultative Heterofermentor	MH544771 MH544781 MH544792		
	*P. pentosaceous* (3)	Facultative Heterofermentor	MH544762 MH544770 MH544778		
	*Lb. paraplantarum* (1)	Facultative Heterofermentor	MH544777		
	*Lb. sakei* (1)	Facultative Heterofermentor	MH544786		
Real (6 from MRS5)	*P. pentosaceous* (2)	Facultative Heterofermentor	MH544765 MH544774	Not Detected	
	*E. mundtii* (1)	Homofermentor	MH544788		
	*Lb. plantarum* (1)	Facultative Heterofermentor	MH544785		
	*E. faecium* (2)	Homofermentor	MH544776 MH544796		
Mixed (5 from MRS5)	*P. pentosaceous* (2)	Facultative Heterofermentor	MH544768 MH544787	Not Detected	
	*Lb. graminis* (2)	Facultative Heterofermentor	MH544758 MH544784		
	*Lb. lactis* (1)	Homofermentor	MH544782		
Commercial (5 from MRS5)	*Lb. graminis* (1)	Facultative Heterofermentor	MH544767	Not Detected	
	*P. pentosaceous* (3)	Facultative Heterofermentor	MH544769 MH544772 MH544789		
	*E. mundtii* (1)	Homofermentor	MH544803		
Gluten free (14 from MRS5 and 7 from YMA)	*Lb. graminis* (1)	Facultative Heterofermentor	MH544784	*Wickerhamomyces anomalus* (2)	MH544826 MH544830
	*Lb. plantarum* (3)	Facultative Heterofermentor	MH544777 MH544799 MH544804		
	*P. pentosaceous* (5)	Facultative Heterofermentor	MH544764 MH544766 MH544780 MH544793 MH544798	*Pichia kudriavzevii* (3)	MH544827 MH544828 MH544831
	*L. kimchi* (4)	Facultative Heterofermentor	MH544775 MH544805 MH544773 MH544763		
	*Lb. paraplantarum* (1)	Facultative Heterofermentor	MH544794	*Saccharomyces cerevisiae* (2)	MH544835 MH544833

**Table 4:** Production of exopolysaccharides in sterile quinoa flour fermentation broth supplemented with various sugars by pure cultures of lactic acid bacteria and yeasts.

Microorganism	Quinoa Flour	Carbohydrate Source/Yield			
		Sucrose	Fructose	Lactose	Starch
*P. pentosaceus*	Red	+/2%	+/2%	+/0.5%	+/2%
*P. pentosaceus*	Real	+/2%	+/2%	+/0.5%	+/2%
*P. pentosaceus*	Black	+/2%	+/2%	+/0.5%	+/2%
*Lb. graminis*	Black	-	-	-	-
*Lb. paraplantarum*	Black	-	-	-	-
*Lb. plantarum*	Black	+/1.5%	+/1.5%	-	-
*Lb. plantarum*	Red	+	+	-	-
*Lb. sakei*	Black	-	-	-	-
*Lb. sakei*	Red	-	-	-	-
*L. lactis*	Black	+/1.8%	+/1.9%	+/2%	-
*E. faecium*	Red	+/1%	-	-	-
*E. faceium*	Real	+/1%	-	-	-
*Le. citreum*	Black	+/1%	-	-	+/1%
*Le. mesenteroides*	Red	+/1%	-	-	-
*W. cibaria*	Red	+/1%	-	+/1%	+/1%
**Yeasts**					
*S. servazzii*	Red	+/<1%	+/<1%	-	+/<1%
*S. servazzii*	Black	+/<1%	+/<1%	+/<1%	+/<1%

The production of ropy colonies is identified with a plus sign (+); while lack of the phenotype is conveyed by a minus sign (−).

**Table 5:** Colony counts (log colony forming units (CFU)/mL), pH and TTA observed from pure cultures of LAB in the fermentation broth at 8 and 18 h.

Microorganism	Source	8 h			18 h		
		log CFU/mL	pH	%TTA	log CFU/mL	pH	%TTA
Control		BDL	5.95 ± 0.02	1.50 ± 0.01	BDL	5.75 ± 0.01	1.80 ± 0.02
*E. faecium*	Real	8.13 ± 0.02	4.83 ± 0.01	2.21 ± 0.02	9.14 ± 0.01	3.33 ± 0.02	4.98 ± 0.05
*E. faecium*	Red	8.33 ± 0.01	5.01 ± 0.01	2.15 ± 0.03	9.25 ± 0.03	3.33 ± 0.01	5475 ± 0.01
*L. lactis*	Black	3.20 ± 0.03	5.62 ± 0.03	1.53 ± 0.04	BDL	5.35 ± 0.04	2.55 ± 0.03
*Lb. graminis*	Black	3.30 ± 0.02	5.31 ± 0.02	1.65 ± 0.03	BDL	5.02 ± 0.05	2.52 ± 0.03
*Lb. paraplantarum*	Black	4.24 ± 0.01	5.95 ± 0.04	1.56 ± 0.01	1.21 ± 0.04	4.99 ± 0.02	2.71 ± 0.02
*Lb. plantarum*	Red	5.38 ± 0.02	5.26 ± 0.02	1.61 ± 0.02	4.32 ± 0.03	4.26 ± 0.01	2.74 ± 0.03
*Lb. plantarum*	Black	5.38 ± 0.01	5.26 ± 0.01	1.75 ± 0.01	4.2 ± 0.01	4.26 ± 0.03	2.61 ± 0.03
*Lb. sakei*	Black	3.30 ± 0.01	5.31 ± 0.01	1.57 ± 0.02	BDL	5.04 ± 0.02	2.51± 0.04
*Lb. sakei*	Red	3.10 ± 0.02	5.24 ± 0.04	1.56 ± 0.01	BDL	5.03 ± 0.04	2.52 ± 0.03
*Le. citreum*	Black	7.40 ± 0.02	4.50 ± 0.05	2.51 ± 0.02	8.40 ± 0.02	3.40 ± 0.01	5.01 ± 0.02
*Le. mesenteroides*	Red	8.14 ± 0.03	4.99 ± 0.01	2.27 ± 0.01	8.92 ± 0.02	3.99 ± 0.01	4.87 ± 0.01
*P. pentosaceus*	Red	8.07 ± 0.01	4.99 ± 0.03	2.25 ± 0.02	8.92 ± 0.03	3.60 ± 0.02	4.85 ± 0.01
*P. pentosaceus*	Black	8.67 ± 0.01	4.57 ± 0.01	2.39 ± 0.01	8.67 ± 0.01	3.12 ± 0.03	4.89 ± 0.03
*P. pentosaceus*	Real	7.94 ± 0.01	4.38 ± 0.02	2.74 ± 0.01	7.94 ± 0.01	3.57 ± 0.04	5.02 ± 0.01
*W. cibaria*	Red	7.35 ± 0.02	4.50 ± 0.01	2.43 ± 0.03	8.35 ± 0.01	3.41 ± 0.03	5.01 ± 0.01

Data shown represent the mean values ± standard deviation of triplicate measurements. A *p*-value of less or equal to 0.05 was applied for the determination of statistical significance. The control was quinoa flour not subjected to fermentation. BDL abbreviates below detection limit.

**Table 6:** Fermentative profile of selected isolates cultivated in sterile flour after 8 h of fermentation.

Microorganism	Quinoa	Lactic Acid (mg/g)	Acetic Acid (mg/g)	FQ
Control		BDL	BDL	−
*E. faecium*	Real	6.47 ± 0.02	0.67 ± 0.03	9.66
*E. faecium*	Red	6.34 ± 0.02	0.82 ± 0.01	7.73
*L. lactis*	Black	1.36 ± 0.01	BDL	−
*Lb. graminis*	Black	1.45 ± 0.01	BDL	−
*Lb. paraplantarum*	Black	3.37 ± 0.01	0.34 ± 0.01	9.91
*Lb. plantarum*	Red	1.88 ± 0.03	0.37 ± 0.01	5.08
*Lb. plantarum*	Black	1.32 ± 0.01	0.39 ± 0.01	3.38
*Lb. sakei*	Black	1.35 ± 0.02	0.25 ± 0.02	5.40
*Lb. sakei*	Red	1.44 ± 0.02	0.28 ± 0.02	5.14
*Le. citreum*	Black	3.21 ± 0.01	0.87 ±0.01	3.69
*Le. mesenteroides*	Red	5.63 ± 0.01	0.70 ± 0.02	8.04
*P. pentosaceus*	Red	2.75 ± 0.01	0.68 ± 0.03	4.04
*P. pentosaceus*	Black	2.66 ± 0.01	0.65 ± 0.01	4.09
*P. pentosaceus*	Real	2.34 ± 0.02	0.71 ± 0.04	3.30
*W. cibaria*	Red	2.01 ± 0.02	0.61 ± 0.05	3.30

BDL: Below detection limit. FQ: Fermentation quotient. Results indicate mean values ± standard deviation of three measurements (carried out in duplicates for two independent fermentations). *p*-value less or equal to 0.05.

## Supplementary Materials

The following are available online at https://www.mdpi.com/2304-8158/9/3/337/s1, Table S1: Chao1 and no. of observed OTUs for the ITS region amplicon sequences corresponding to the fungi present in quinoa flour fermentations using the 454 Sequencing Technology. Sequencing data samples were rarefied to 7000 reads for the Alpha Diversity Chao1 analysis, which only included samples containing more than 1000 reads.
