# Supplementary material for: Isolation of Exopolysaccharide-Producing Yeast and Lactic Acid Bacteria from Quinoa (Chenopodium Quinoa) Sourdough Fermentation
Source: Foods. 2020 Mar 13;9(3):337. doi: 10.3390/foods9030337 (PMC7142942; doi:10.3390/foods9030337)
Supplement: Supplementary file 1 [file foods-09-00337-s001.pdf]

Table S1. Chao1 and no. of observed OTUs for the ITS region amplicon sequences corresponding to the fungi present in quinoa flour fermentations using the 454 Sequencing Technology. Sequencing data samples were rarefied to 7000 reads for the Alpha Diversity Chao1 analysis, which only included samples containing more than 1000 reads.

Quinoa Flour Type / Replicate	Fermentation Time (d)	Chao1	Observed OTUs
Red / Rep 1	6	9.1 ± 0.1	2.4 ± 0.3
	8	6.2 ± 0.1	9.1 ± 0.1
Red / Rep 2	1	3.8 ± 0.3	6.2 ±0.1
	6	3.0 ± 0.1	3.8 ± 0.3
	8	3.0 ± 0.1	3.0 ±0.0
Black / Rep 1	1	3.9 ± 0.5	5.0 ± 0.1
	4	1.0 ± 0.0	3.7 ± 0.4
	6	3.0 ± 0.0	3.0 ± 0.1
	8	2.7 ± 0.3	3.0 ± 0.0
Black / Rep 2	1	7.9 ± 0.3	3.0 ± 0.1
	4	2.4 ± 0.3	7.9 ± 0.4
	6	3.2 ± 0.1	2.7 ± 0.3
	8	5.0 ± 0.1	5.0 ± 0.3
